# Analysis of Bacterial Communities around the Adventdalen Landfill Site in Svalbard

**DOI:** 10.3390/microorganisms11041093

**Published:** 2023-04-21

**Authors:** Hermi Amores-Arrocha, Alex K. B. Asamoah-Asare, Joyce Opio, Alex Martin, Lewis Cuthbertson, Hannah R. Bradford, Maria-Luisa Avila-Jimenez, David A. Pearce

**Affiliations:** 1 Department of Applied Sciences, Faculty of Health and Life Sciences, Northumbria University at Newcastle, Northumberland Road, Newcastle-upon-Tyne NE1 8ST, UK; hermiamar@gmail.com (H.A.-A.); boma.asare@gmail.com (A.K.B.A.-A.); joyce.opio@onet.pl (J.O.); alexj.martin@outlook.com (A.M.); lewispc1993@icloud.com (L.C.); hannahrainebradford@hotmail.co.uk (H.R.B.); 2 Nature Metrics, Guildford GU2 7HJ, UK; malu.avila@naturemetrics.co.uk

**Keywords:** Arctic, bacteria, landfill, resilience, diversity

## Abstract

Ecosystems are often resilient enough to fully recover following a natural disturbance, or to transform into a new equilibrium favourable to the surrounding flora and fauna. However, at a local level, whether this transformation will be beneficial or not depends strongly on the level of disturbance and the available mechanisms for recovery. The Arctic, however, provides a potentially extreme environment for microbial growth and this is reflected in the microbial biodiversity, the in-situ growth rates, the biogeochemical cycling and its sensitivity to environmental change. In this study, we evaluated the current microbial biodiversity and environmental conditions around the landfill site in Adventdalen, Svalbard to identify differences across bacterial communities that might promote or accelerate naturally occurring environmental recovery. Landfill sites can induce changes in the local environment through the input of exogenous chemicals (both organic and inorganic) and microorganisms. Leachate can flow with run-off from the primary location of the landfill site due to rain, snow or ice melt and spread material into soils surrounding the site. In this study we found a strong effect of the landfill site on the bacterial diversity in the local landscape. Intervention is highly desirable to enhance the environment and improve the restoration by subtly altering the conditions at the site (such as the pH or drainage courses) and by encouraging specific groups of naturally occurring indigenous microorganisms to bioremediate the site.

## 1. Introduction

Soils and sediments are the cornerstone of the Earth’s biogeochemical cycles, and the microbial communities they contain are essential to maintain the water–soil–atmosphere equilibrium, as exemplified by the current increase in atmospheric CO_2_ associated with the melting of the tundra due to climate change [1]. Soils host highly diverse microbial communities at relatively high biomasses, and mediate essential processes such as the nitrogen, phosphorus and carbon cycles. In general, these environments are thought to be relatively robust and adaptable to gradual environmental change. However, above a critical threshold, the soil can entirely lose its ability to recover from such disturbances, leading to long-term changes with often unpredictable consequences [2,3,4,5,6]. This is potentially more so for the slow-functioning Arctic environments disturbed by anthropogenic activities, such as landfill sites. However, environmental engineering is able to exploit microbial communities already naturally occurring in the environment to potentially revert this situation, increasing the possibility of a full recovery of the natural habitat following a disturbance. A key example is the promotion of harmless oil-degrading bacteria to deal with the consequences of oil spills [7]. In this study, we sought to evaluate the microbiological status of one such landfill site in Adventdalen on the headland between Todalen and Bolterdalen in Svalbard, in the Norwegian Arctic, to identify the potential future actions that might promote naturally occurring microbial communities to accelerate environmental recovery. To do this, we combined molecular analyses of soil bacterial communities, characterised the nature of the environmental changes that have been induced at the landfill site, determined whether any intervention is necessary (or desirable) to restore the original environmental conditions and attempted to evaluate whether the area of influence of the landfill site is stable, receding or increasing over time.

Arctic and boreal environments cover ~22% of the terrestrial surface of the planet and are very sensitive to environmental change—both natural perturbations and those which result from human activity. The Arctic provides a potentially extreme environment for microbial growth, and this is reflected in the microbial biodiversity, the in-situ growth rates, the biogeochemical cycling and its sensitivity to environmental change. A number of local factors are responsible for this, including the arid climate, extreme low temperatures and varying light and UV exposure. In Svalbard (78° North), the temperatures in winter are on average −20 °C, in the summer they average +6 °C [8] and during the transition periods there is regular freeze–thaw cycling, often at a relatively high frequency. The polar night, consisting of 24 h of darkness lasts from the end of October to mid-February, limiting growth among phototrophs, which already could be slow in psychrophilic conditions. In contrast, during the summer, mid-April to the end of August, the polar day brings 24 h of sunlight. The warmer climate and high levels of solar radiation can lead to snow melt and the thawing of ice within the ground.

Research interest in the Arctic environment is increasing due to the extreme environmental conditions and the rate of climate change [9]. Global warming poses a significant threat to the Arctic tundra as it has been observed to cause the melting of permafrost, which in turn, will likely affect the species that survive or colonise the area. The low temperatures in polar soils typify a slow-functioning microbial community which can be more susceptible to anthropogenic activities. Indeed, the polar regions potentially represent some of the most vulnerable ecosystems that could be affected by frequent perturbations and relatively long-term climate change [1]. Although there is limited research that specifically addresses the impacts of human activity on both the Arctic and Antarctic [10,11], it has become evident that biota in these regions are experiencing significant environmental change. According to the IPCC, global warming is predicted to occur rapidly and to its greatest extent in areas of high latitude, particularly the Arctic [1]. 

The biodiversity of tundra ecosystems is generally low due to the harsh climate with a bacterial population density that is relatively low for Arctic soils compared to temperate soils [12]. Organisms in the Arctic are affected by environmental changes that occur not only as a result of climate change but also due to the effects of human activities ranging from tourism, power generation, mining activities and even infrastructure development [13].

The bacterial community composition in soil can change significantly in response to environmental change [14], although specific detail is lacking [15]. The structure and diversity of soil bacterial communities have been found to correlate with both the pH [16] and other soil environmental variables [17]. However, other soil characteristics such as the nutrient availability, cationic metal solubility, organic carbon, soil moisture regimen and salinity are often found to be directly or indirectly correlated to the soil pH. For example, the apparent influence of the soil pH makes it a good predictor of the likely changes in the community structure for the Acidobacteria and Actinobacteria [18]. In addition, the abundance of the Bacteroidetes, Betaproteobacteria and Acidobacteria can be related to carbon availability as the Firmicutes are dominant for cellulose decomposition in landfills irrespective of location [15]. Although the pH appears to be a driver of many of the patterns in soil microbial diversity, the influence of other factors may predict the soil microbial community structure across larger spatial scales. As Chu et al. [19] reported, the pH may not directly alter the bacterial community structure but rather impose a physiological constraint on soil bacteria, such as the Acidobacteria, altering competitive outcomes and reducing net growth when the soil pH falls outside a critical range [19]. Further, the proteobacteria taxa are not well correlated with pH, suggesting that the abundance of these groups are predominantly influenced by factors other than the pH. Many bacteria have intracellular pH levels close to neutral, and therefore, the extreme pH may impose significant stress.

### 1.1. Landfill Sites

Landfill sites have been a common method for disposing of domestic waste for centuries and are the most widely employed methods across the globe [20,21,22]. This practice can lead to a significant build-up of refuse. However, the build-up of refuse can also inadvertently contaminate the surrounding environment through surface run-off and affect the quality of the surrounding water and soil [21,23,24,25,26]. Run-off is mainly caused either directly as water from precipitation as snow and rain infiltrates the landfill site from above or indirectly from below the site via surface flow. Once inside, water accumulates both biological and chemical substances which can then leach out of the site. This leachate may potentially be highly mineralised, as some of the material may not be fully degraded or removed by other means, and then flow out of the site into the surrounding area [22,27]. The composition and level of contamination will depend on various factors such as the amount of rainfall, the age of the landfill site, the waste composition and the degradation stage of the waste [21,28,29]. However, it is possible to study the decomposition processes within and around a landfill site. For example, through the use of PCR based molecular studies of landfill leachate, researchers directly detected bacterial species involved in the degradation of cellulose, the primary carbon source in most landfills [30]. The composition of the generic landfill leachate can be divided into four main categories: (1) dissolved organic matter which contains alcohols, acids, carbohydrates, etc., (2) inorganic macro components such as common cations and anions like sulphur, chlorines, ammonia, etc., (3) heavy metals such as iron, lead, nickel, copper, chromium, etc. and (4) xenobiotic organic compounds which include antibiotics, drugs and other compounds such as polychlorinated dibenzodioxins (PCDD) [31]. A study based on more than 70 municipal solid waste landfills in both Europe and the United States found that the chemical composition of landfill leachate contained many different chemicals, and that as the landfill ages, the concentrations of these chemicals decreases [22,32]. In general, landfill degradation occurs in three phases. To begin with, there is aerobic degeneration due to the ready availability of oxygen. The next phase is anaerobic degradation, which decreases the leachate pH, then methanogens reduce the carbon dioxide and hydrogen into methane. This decrease in the pH causes some chemicals, such as aluminium, to become more soluble, and therefore, more toxic. Organic compound concentrations also show a notable decrease. As acids produced during the decomposition are consumed, the pH of the site becomes relatively neutral [31,33].

### 1.2. Acid Mine Drainage (AMD)

Another contributory factor to the pH story is acid mine drainage (AMD). The Adventdalen landfill site is located on top of an area of an acid mine drainage impact. Indeed, the site was originally chosen due to the effects and impact already seen at that location. In general, acidic sulphur-rich wastewater is produced by industrial operations. However, the most common cause is from the mining industry. The water draining from both active and abandoned mines is often extremely acidic. The low pH increases the solubility of the transition metals causing the drainage to contain elevated metal concentrations, such as iron, manganese and aluminium, with the potential for other harmful heavy metals such as arsenic [34,35]. Acid mine drainage has a profound effect on the local biodiversity. The influence of acidic, metal-rich fluid causes a shift in the soil pH, preventing the growth of bacteria that cannot adapt to the acidic environment. There is also an effect of heavy metals that flow from the mine that may cause toxicity in some instances. Trace metals released in the acid mine drainage can include highly toxic metals, such as nickel and copper, as well as many other harmful trace metals, such as cobalt and lead. It has also been shown that most metals, with the exception of iron, show a negative correlation with the pH, meaning that the higher the pH, the lower the concentration of metals present [36]. Aside from the toxic effects of the elevated levels of metals in solution, particularly iron and aluminium, the metals will also potentially accumulate in the sediment surfaces and can interfere with aquatic life cycles. AMD is not only associated with surface and groundwater pollution but is also responsible for the degradation of the surrounding soils. 

### 1.3. Arctic Microorganisms and Their Relevance

Studying microbial processes in the Arctic is still in its relative infancy but it is crucial as this region responds rapidly to environmental change and is susceptible to climatic control. There is also considerable potential in directly using the microorganisms from the polar regions in bioremediation studies to neutralise or eliminate the pollutants from a contaminated site, resulting in non-toxic or less toxic products [37,38], for example, via the alpha-proteobacteria or the Actinobacterium *Rhodococcus* sp. [39]. 

Bacterial diversity in the polar regions has been found to be dominated by few bacterial phyla. In general, these are the alpha-, beta- and gamma-proteobacteria, the Cytophaga-Flavobacterium-Bacteroides group and high GC Gram-positive phyla. Other dominant groups in polar regions include Actinobacteria such as the *Nocardia* sp. and *Mycobacterium* sp., and Gram-negative bacteria such as *Pseudomonas* sp. and *Spirosoma* sp. [17,40,41]. Cyanobacteria comprise a large part of the microbial community due to their inherent resistance to harsh environmental conditions. Terrestrial Arctic environments also generally have low nutrient levels, allowing Cyanobacteria to dominate some habitats due to the nitrogen fixation properties of some species [42]. 

The bacterial community composition is also linked to the vegetation type, geographical region, quality of soil organic matter and environmental factors that include but are not limited to the temperature, soil pH, water and nutrient availability [43]. The warmer temperatures in the Arctic could be important due to increases in plant growth and its consequent increase in substrate provision [44]. Irrespective of the harsh soil conditions that include low water activity, protracted subzero temperatures and limited nutrient availability, molecular biology investigations carried out in the cold terrestrial habitats of the Arctic have estimated a high microbial biomass and diversity, with up to 10^9^ bacteria per gram of soil [45]. The prominent groups that have been identified belong to the phyla proteobacteria, Actinobacteria, Acidobacteria, and to a lesser extent, Bacteroidetes and Firmicutes [44,45,46,47]. Liebner et al. [45] provided insight into the functional microbial groups in the Arctic, such as methanotrophic bacteria and methanogenic archaea, which, despite being studied quite extensively, not yet in specific relation to the stability of the bacterial community structure or ecosystem change. It is noted that even with thick snow or ice cover of the soil in Arctic areas, the temperatures within the soil remain close to 0 °C, allowing for the continuity of microbial activity, arguably where the Acidobacteria and proteobacteria species tend to dominate [48].

Microbial activity can help curb the impact and rate of environmental pollution as a result of the metabolic processes of the Arctic soil bacteria breaking down hydrocarbon build-up and releasing elements such as methane and nitrogen. However, these organisms have systems that are highly temperature dependent or regulated. The lower temperatures that exist in the Arctic areas, therefore, can slow the rate at which these bacteria can clean up potential contaminants [49]. The polar areas are, however, useful places to investigate the process of bioremediation, particularly under extreme and changing conditions. This opportunity is aided by the very fragile nature of polar soils and the long periods of time they require to recover from any effects of potential pollution [50]. 

The aim of this study was to analyse both the organic and inorganic compounds in soil samples taken from the Adventdalen landfill site to determine any change in the environment that could have resulted from leachate from the site and to investigate its potential influence on the attendant microbial community. 

## 2. Materials and Methods

### 2.1. Site Description

**Figure 1:**
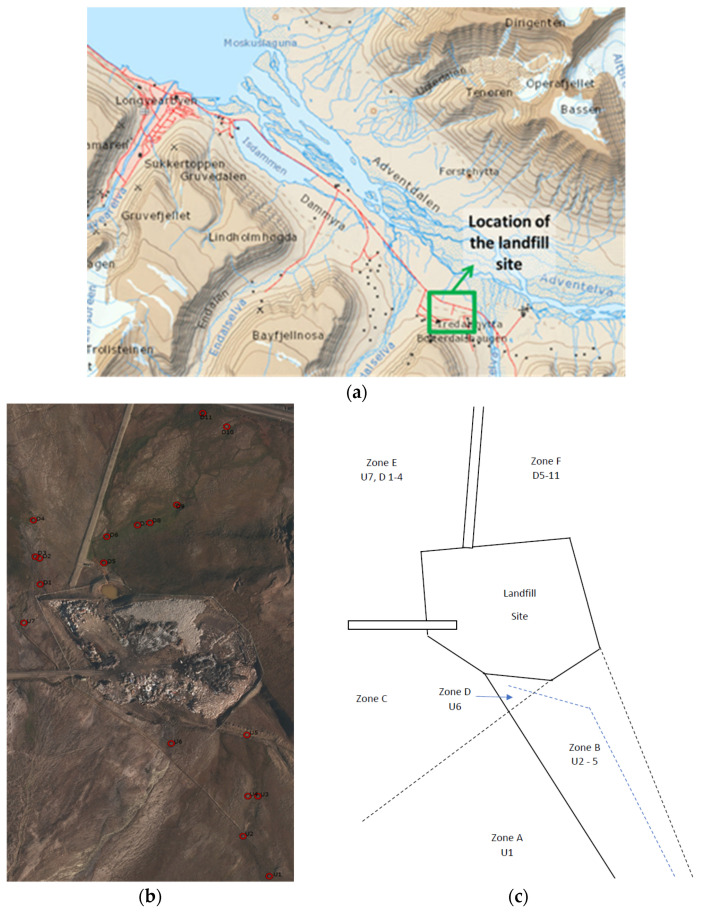
(**a**) the location of the landfill site on Svalbard (Longyearbyen to the landfill site is ~10 km), (**b**) an aerial view of the sample locations and (**c**) a schematic showing the position of the different zones.

The Arctic includes a large area of land, sea and ice covering approximately 40 million km^2^ [51,52]. This study took place in Svalbard, 1000 km from the North Pole between 74°–81° North and 10°–35° East and with a total land area of 62,400 km^2^. The mean air temperatures in the Svalbard region within the warmest month (covering three zones—the mid-Arctic tundra zone, northern Arctic tundra zone and polar desert zone) range from 1 °C to 6 °C [53]. In winter, however, the temperatures ranges from 0 °C to −40 °C [54]. The Arctic landscape is predominantly covered with tundra, rocks and bare soil or ice/snow. It has permafrost soil that inhibits tree growth. The vegetation usually comprises grasses, mosses, sedges, herbs, lichens and dwarf shrubs that thrive in short growing seasons, annually cold temperatures and permanently frozen soil conditions [52]. The Adventdalen landfill site is located roughly 10 km from Longyearbyen, where the main population of Svalbard is concentrated (Figure 1a). The landfill was established in 1991 with a life expectancy of 75 years. A report on the site published in 2011 showed that in 2001, approximately 1000 tons of waste had been deposited. By 2006, this had increased to 1700 tons. After 2007, municipal waste was no longer deposited at the landfill site and it was then used mainly for inert masses or non-degradable waste, such as gypsum, plasterboard, plastic, steel, concrete, insulation materials, glass etc. [55,56]. In Longyearbyen, municipal waste is now incinerated or returned to mainland Norway rather than being deposited in the landfill [57]. However, there are still likely remnants of municipal waste from before 2007 at the Adventdalen site. In addition, while active, solid waste and ash from the coal fired power station were also deposited at the site, which may contain traces of heavy metals, PAHs and dioxins [57]. Most of the landfills in Longyearbyen rest on old rubbish, and hence, are dumpsites rather than sanitary landfills. The presence of the now unused coal mine near the site may potentially contaminate the surrounding area around the landfill with continuous acid mine drainage, even though the site itself is no longer used. This will likely cause further changes to the surrounding environment and its microbial communities as the climate warms.

The main landfill site has three streams originating from different melt sources Figure 1a–c). At the time of main sampling (9 July 2015), the conditions were sunny with little cloud cover and an air temperature of 10.4 °C. There had also been very little rain to contribute to the melt streams. One stream originating from the south-west came from an area with minimal obvious influence from human factors with pH values ranging from 5.7–6.2 (Zone A and B, Figure 1c). To the edge of this stream, there was a large wet area which appeared to be caused by flooding of the original stream leaving some stagnant ponds, which were found to be warmer than the main stream and with a more neutral pH of 6.5. This stream also appeared to have been altered in order to bypass the landfill via a an embankment where it was added to by a second stream originating from the mountain to the south-west (Zone C). This water originated from glacial melt on the mountain located next to an unused coal mine which then ran down the mountain and was added to the water in the bypass. Due to the proximity of the mine, it was observed that at this meeting point the stream overflowed and the water entered the landfill (Zone D). Samples were taken at this point (U6, Figure 1b) and, due to the influence of the mine, the samples had a very acidic pH (of 3.4). This bypass continued past the landfill and then to the north-west to a larger body of water near a road (Zone E). There was evidence of potential contamination from a possible stream originating from the north-west corner of the landfill and entering this stream. The final potential stream was dry on the day of sampling. However, a sample was taken (U5, Figure 1b) as the stream entered the landfill itself and was unaffected by the bypass stream. The stream from the landfill site originated from a large pool of water to the East side of the site and then out of the site and into the land to the south-east (Zone F). The stream continued down towards the road. Surrounding the stream were several stagnant ponds. Plenty of vegetation was evident and wildlife was seen grazing in the area. For comparative purposes, the site was divided into upstream ‘U’ (Zones A–D) and downstream ‘D’ (Zones E and F) sectors.

### 2.2. Sample Collection

Initially, nine samples were taken of the surface sediment (SS) 100 m and 200 m upstream of the landfill site for SS1, SS2 and SS3 and SS4, SS5 and SS6, respectively, and 50 m downstream for SS7, SS8 and SS9 to investigate whether differences in the microbial biodiversity could be detected upstream vs. downstream of the site (Year 1, 2014—upstream vs. downstream). To increase the spatial resolution and investigate patterns in the chemical composition, a further 18 soil sediment samples were then collected the following year on 9 July 2015 (Year 2, 2015—transect). These samples were identified as upstream (U) 1–7 and downstream (D) 1–11 of the landfill site. The upstream sites were sampled at different points on the surrounding streams that appeared to enter the landfill from various sources. The downstream samples were also taken for comparison to the upstream samples to see what influence the landfill site had on the pH, temperature and both organic and inorganic chemical composition. Samples D1–D4 were taken from the floodplain downstream of the bypass stream with influence from the water current from the north-west bend (Zone E). The samples D5–D11 were taken downstream of a stream that flowed directly from the landfill itself (Zone F). The samples were placed into sterile 250 mL wide neck plastic bottles and sealed. At the time of sampling, the pH and temperature of both the soil and water were taken and recorded. The sample map can be found in Figure 1b.

### 2.3. DAPI (4′,6-Diamidino-2-Phenylindole) Total Cell Counts

For this study, 5 g of sediment sample was diluted 1:10 in 0.2 µm filtered sterile Milli-Q water and phosphate buffered saline PBS (PBS; 10 mM sodium phosphate [pH7.2], 130 mM NaCl [pH7.4]), and then homogenised briefly using a vortex mixer. The cells were harvested using vacuum filtration (~30 kPa) through a 25 mm, 0.22 µm, black, polycarbonate filter membrane. The filters were then stained with DAPI (5 mg ml^−1^) for 5 min and rinsed twice with sterile Milli-Q water and PBS (5 mL). The bacterial cells were counted using an epifluorescence microscope equipped with a DAPI filter and recorded. Due to the movement of water across the site, additional samples were taken at D13 and D14 in the stagnant ponds next to the stream where there was no water movement. Samples D16 and D17 were also taken at the end of the stream flowing from the landfill at the road. 

### 2.4. DNA Extraction and Sequencing

Samples were taken from the top of the soil using clean trowels or scoops, placed in sterile, non-cytotoxic, non-pyrogenic Falcon™ tubes and stored in a refrigerator or on ice at −20 °C. Approx. 1–2 g of the soil/sediment samples were taken and placed in separate 2 mL microcentrifuge tubes and sealed. The extraction was carried out using the PowerSoil^®^ DNA Isolation kit (Qiagen, Germantown, MD, USA), following the manufacturer’s instructions. The extracted DNA was purified and concentrated directly using Illustra GFX PCR DNA and Gel Band Purification kits (GE Healthcare, Chicago, IL, USA), and following the manufacturers’ instructions. These tubes were then sent for 16S rRNA gene sequencing at the MrDNA laboratory in Shallowater, Texas. The sequence data were analysed using QIIME and Mothur software and a principal component analysis was conducted on the data to determine differences in community profiles for each sample site.

### 2.5. pH

The pH was directly determined using a hand-held portable pH meter (VWR pH10 Pen, VWR International, Radnor, PA, USA) in the field, due to its influence on the solubility of metals.

### 2.6. X-ray Fluorescence (XRF)

The inorganic elements were determined using X-ray fluorescence (XRF). Sediment samples were weighed in 50 mL falcon tubes for drying, allowing initial calculations of the percentage water loss. The samples were then placed in a drying cupboard until completely dry. Dry samples were then milled into a fine powder using a Frich Pulverisette 6 Ball Mill. Approx. 4 g of this fine soil was mixed with 0.7 g of a Cereox binder, with both weights being measured to three decimal places. Tubes containing the soil-binder mix were then placed on an electronic shaker for 3 min until fully mixed. The mixture was fashioned into an XRF pellet using a manual desktop hydraulic press at 10,000 kg. This procedure was repeated until there were duplicate pellets from each sampling site. The pellets were then labelled and analysed using a Spectro X-Lab 2000. The duplicate measurements were averaged. 

### 2.7. Total Organic Carbon (TOC) and Organic Chemistry (GC-MS)

Total organic carbon was determined using the Walkley-Black method. Approx. 400 µg of replicate dry sediment samples were added separately to 1 L Erlenmeyer flasks. The flasks were then moved into a fume cupboard with heat mats, where 10 mL of potassium dichromate (K_2_Cr_2_O_7_) was added using an automatic burette. Following this step, 20 mL of sulphuric acid (H_2_SO_4_) was added and the flasks were left to cool for 45 min atop the heat-proof surface. The amount of dichromate added to each sample was noted for the final calculation. Then, 170 mL of distilled water and 10 mL phosphoric acid was added to each flask. Two to three drops of diphenylamine indicator were then added to each flask before titration. Each sample was titrated against 0.5 M ammonium ferrous sulphate. 

For GC-MS, after defrosting at −3 °C, 1 g of soil was weighed in a glass culture tube and diluted with 4 mL dichloromethane before a 10 min incubation in a Thermo Fisher ultrasonic water bath. 2 mL of the sample was dispensed into a glass filter tube and 12 × 32 chromatography HS vials. The samples were analysed in a Thermo Scientific Trace 1300 gas chromatography machine coupled with ISQ7000 single quadrupole mass spectrometry and an AI13000 autosampler. A helium carrier gas was used. Analysis was performed at a 250 °C injector temperature with a 32-min equilibration. The data output and evaluation was carried out using the Chromeleon™ CDS software.

## 3. Results

### 3.1. DNA Extraction and Sequencing (Year 1—Upstream vs. Downstream Comparison)

The total species number and Shannon diversity index are given in Table 1. 

**Figure 2:**
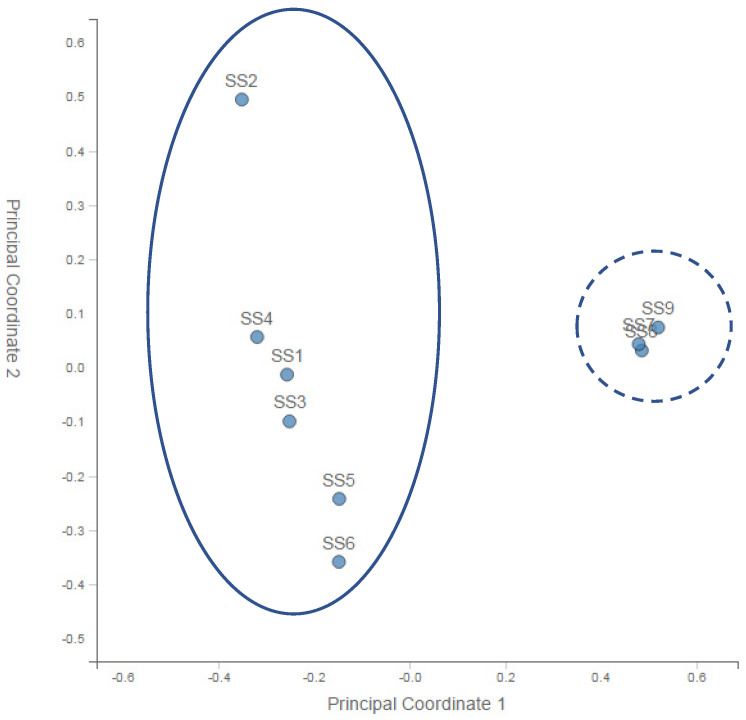
Principal coordinate analysis (PCoA) of the normalised relative abundance of all the preliminary samples at the genus level. SS—substrate sample. Solid line—upstream sites (1–6). Dashed line—downstream sites (8–9).

A principal coordinate analysis (PCoA) of the normalised relative abundance of all the preliminary samples is given in Figure 2. PCA analysis of the community profiles showed clear differences in bacterial community structure between the sample points upstream of the landfill site and a strong correlation among all of the bacterial communities downstream of the landfill site.

**Figure 3:**
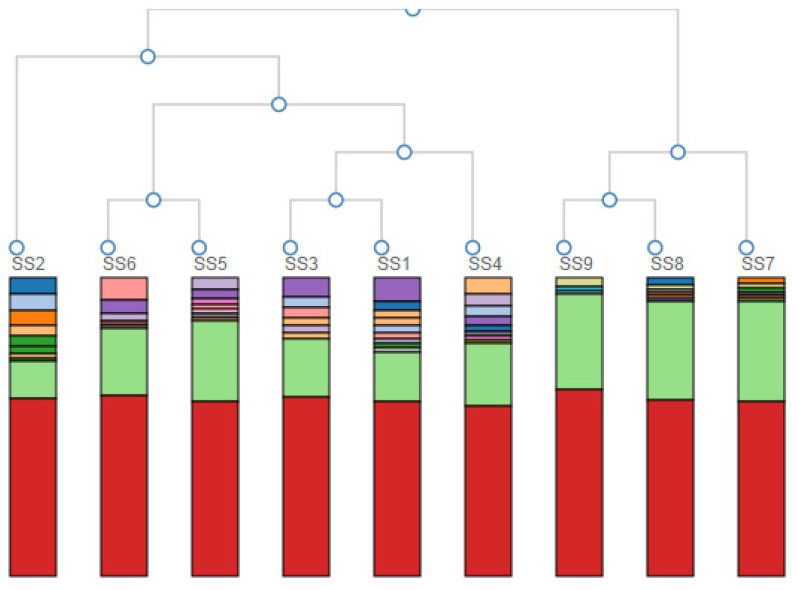
Hierarchical clustering of the samples based on the genus-level classification. Colours represent different Genera—the red colour reads as unidentified at the genus level (around 50%).

A hierarchical clustering of the samples based on the genus-level classification is given in Figure 3. The hierarchical clustering grouped the data over a variety of scales by creating a cluster tree or dendrogram. It involved creating clusters that had a predetermined ordering from top to bottom, creating a multi-level hierarchy.

### 3.2. DAPI (4′,6-Diamidino-2-Phenylindole) Total Cell Counts

The total DAPI counts are presented in Table 2. The sample sites D13 and D14 were taken in the stagnant ponds next to the stream where there was no water movement. Samples D16 and D17 were taken at the end of the stream flowing from the landfill at the road.

### 3.3. pH and Temperature

Duplicate recordings of the pH and temperature of both sediment and water were taken at the time of sampling and the average is presented in Table 3.

### 3.4. DNA Extraction and Sequencing (Year 2—Transect)

**Figure 4:**
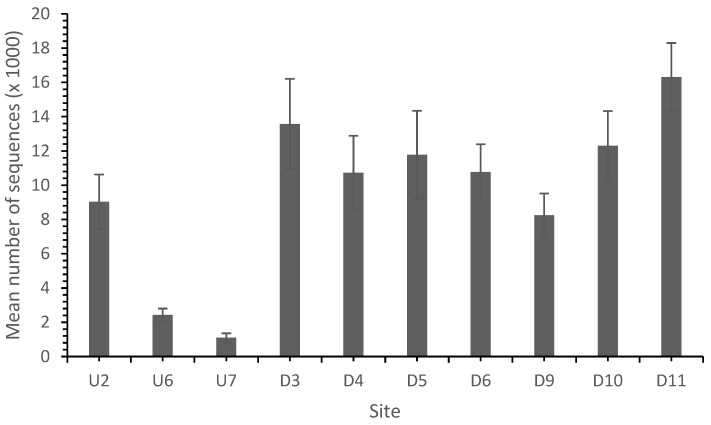
Total sequence number by site. AMD reduced the total sequence number, which recovered as the distance from the landfill site increased.

In common with other studies, the sequence analysis identified bacteria belonging mainly to the phyla proteobacteria, Actinobacteria and Acidobacteria and to a lesser extent Bacteroidetes and Firmicutes [37,41]. In total, 139,725 sequences from 10 sites were identified with a high and variable diversity across the sites (Table 4 and Table 5; Figure 4).

### 3.5. X-ray Fluorescence (XRF)

**Figure 5:**
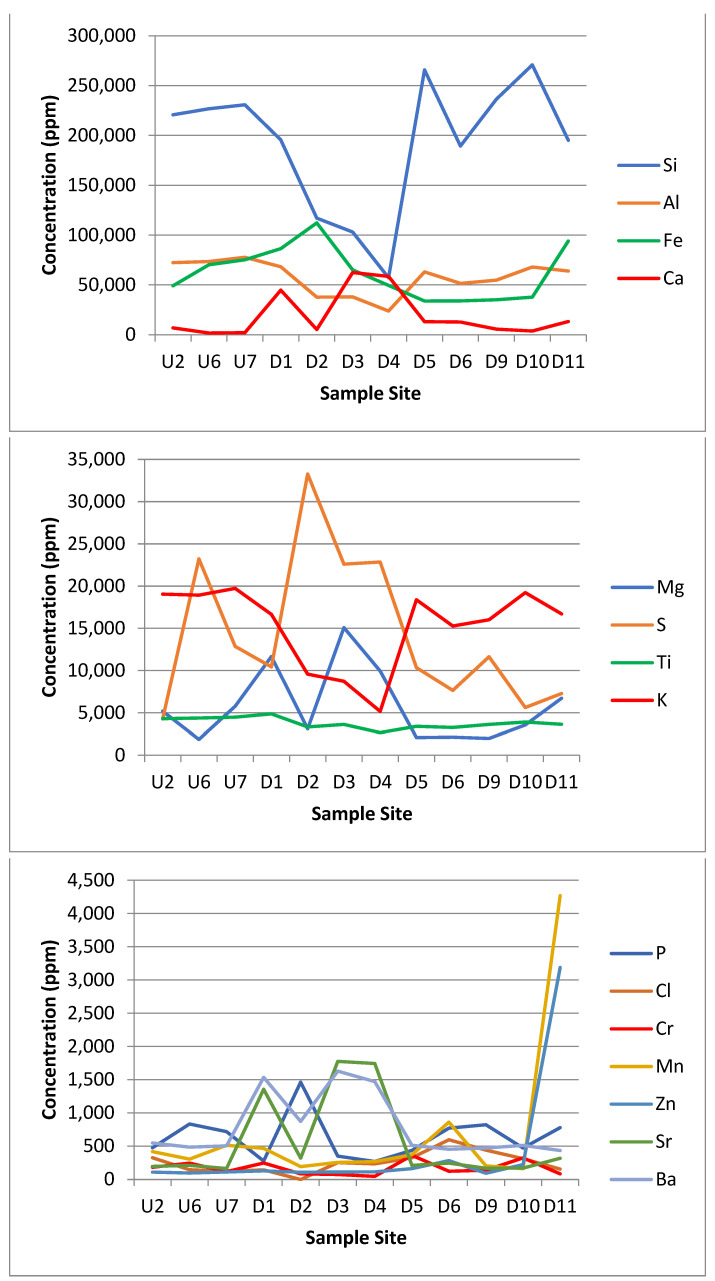

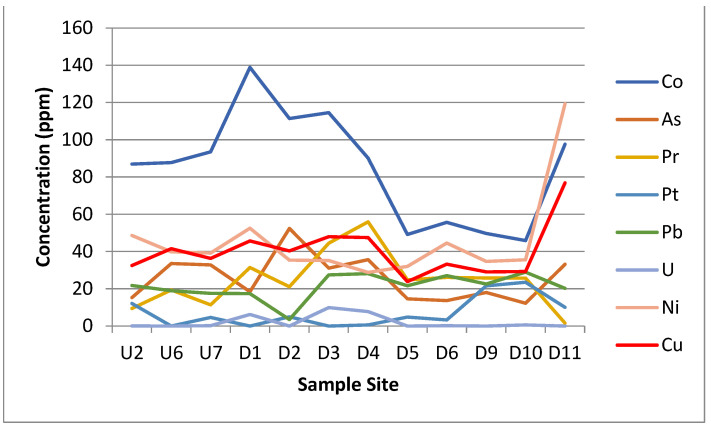
Graphs showing the concentration in parts per million (ppm) of the 23 different elements detected by the XRF. Sample site axis is a proxy for the distance along the transect.

The XRF data for the 23 elements are presented in Table 6 and Figure 5.

### 3.6. Organic Chemistry

The total organic carbon concentrations are given in Table 7. 

In total, 14 major organic compounds were detected (Table 8). The downstream site samples had lower levels of organic chemical diversity compared to the upstream samples, although chemical hotspots (in terms of the hydrocarbon composition) were apparent.

## 4. Discussion

### 4.1. DNA Extraction and Sequencing (Year 1—Upstream vs. Downstream Comparison)

From the nine initial samples taken upstream of the landfill site at 100 m (SS1, SS2, SS3) and 200 m (SS4, SS5, SS6) and downstream at 50 m (SS7, SS8, SS9), it was very clear that differences in microbial biodiversity could be detected across the landfill site. Table 1 showed that across the site, we could expect ~1300 to ~1650 species per sample. This number did not vary systematically across the samples, although SS5 and SS7 (one 200 m upstream and one 50 m downstream) were approximately an order of magnitude lower than this general trend. The reason for this decrease could be the patchy nature of the site as evidenced by the absence and presence of water, the flow rate, the pH, the chemistry and the presence and absence of vegetation. Importantly, looking at the Shannon diversity index (Table 1), it was clear that diversity was highest 50 m downstream of the landfill site, suggesting an increase in the diversity in the landfill leachate. The lowest diversity occurred at a distance upstream of the landfill site, which increased with AMD contact and then increased further after the landfill. This may have reflected different chemical gradients, increased patchiness and/or biodiversity inflows leading to higher levels of niche differentiation. Importantly for this study, the PCA analysis of the community profiles across the site (Figure 2) showed differences in the community structure between the sample points upstream of the landfill site and a strong correlation among all of the communities downstream of the landfill site. Hierarchical clustering of the samples based on the genus-level classification, as shown in Figure 3, also showed a clustering of the downstream samples and some degree of mixing in the upstream samples, suggesting that the landfill site either provided a strong selection pressure for the dominant groups (increasing the overall diversity) or removed all but the most resilient groups (although, in this case, we would expect to see a decrease in the diversity). Overall, it is clear from the year one data that the landfill leachate had a pronounced effect on the bacterial community composition.

### 4.2. DAPI (4′,6-Diamidino-2-Phenylindole) Total Cell Counts

The total cell counts in landfill leachate presented in Table 2 suggested a wide variation in the biomass across the site. Importantly, cells were present even in relatively fast flowing water, suggesting that cells were present and were moved across the site by water flow. Due to the complexity of the variation in flow rates, it was not possible to take this interpretation any further, although additional samples taken from the standing water around the site showed higher cell counts. DAPI counts of bacteria found along the transect showed that the populations were larger in the areas where water movement was slow or barely moving. Results also suggested that there was a clear dilution effect from water movement. Standing water developed a high biomass and often contained biofilms.

### 4.3. Temperature and pH

The temperature was found to vary considerably over the site which may have had some differential effect on bacterial growth. The pH levels both up and downstream of the landfill site were also highly variable, although they could broadly be classified as acidic (under the influence of AMD) or neutral. Samples U6 and U7 were apparently affected byrun-off from the mining site further up the mountain that joined the bypass stream. This stream continued down and resulted in low pH in sample sites D1–4 (Zone E). Compared to this, the sample sites following the landfill stream (Zone F) were all quite close to neutral in both sediment and water (apart from site D8). The neutrality of the downstream samples appeared to be directly caused by the presence of the landfill site itself, likely due to a neutralizing effect of relatively alkaline run-off from the discarded waste. 

This difference in pH could be an important driver of biodiversity. As discussed, the prime importance of soil pH as a control of soil bacterial community structure has been known for some time [14,58,59], and recent studies have shown that bacterial communities in soils from a broad range of ecosystems are strongly structured according to variation in the soil pH [16,17,19,60,61]. By contrast, differences in other soil and site characteristics were poor predictors of bacterial community structure, suggesting that variation in soil organic matter chemistry, vegetation type and environmental factors (other than the soil pH) have relatively small impact on the phylogenetic composition of soil bacterial communities. 

### 4.4. DNA Extraction and Sequencing (Year 2—Transect)

The most striking observation from the transect was the relatively high diversity of bacterial genera across the site. The highest abundance across most sites were the genera *Brevundimonas*, *Devosia*, *Flavobacterium* and *Rhodoferax* (with the exception of U6, U7 and D10, Table 9). In total, 139,725 sequences from 10 sites were observed (Table 4), with variations in diversity across the sites. From the 133 genera identified overall, 15 genera were detected downstream but not upstream of the site, and a further 45 genera identified both upstream and downstream had fewer than 10 species in the upstream sites (much fewer than the same genera in the downstream sites).

The more acidic sites (U2, U6, U7) were dominated by the acidophilic genera *Acidicapsa* and *Acidiphilium* (Table 10). 

The downstream sites in Zone E (D3, D4) were dominated by *Polaromonas* sp., which are ubiquitous in low temperature environments, and *Thiobacillus* sp., which are involved in sulphur cycling, whilst the species belonging to the genus *Ramlibacter* degraded isoprene and/or were methanotrophic (Table 11).

The downstream sites in Zone F (D5, D6, D9, D10, D11) were dominated by the more diverse genera that tended to be more mesophilic, such as *Algoriphagus*, *Alkalibacterium* and *Hydrogenophaga* (Table 12).

Comparing the top four sequence matches across the sites (Table 5), U2 was apparently unimpacted by the landfill site or AMD and was dominated by bacteria we would expect to find in Arctic soils, i.e., *Flavobacterium* sp., *Bradyrhizobium* sp. and *Polaromonas* sp. The AMD influenced low pH sites were dominated by *Terracidiphilus* sp., *Acidophilum* sp., *Acidibrevibacterium* sp. and *Hydrogenophaga* sp., while the landfill leachate sites (Zone F) were dominated by *Rhodoferax* sp., *Bradyrhizobium* sp. and *Polaribacter* sp. *Rhodoferax* sp. are purple non-sulfur bacteria that are frequently found in stagnant aquatic systems exposed to light [62] (also isolated from the Antarctic)*, Bradyrhizobium* sp. are common soil-dwelling microorganisms, some of which can fix nitrogen and *Polarimonas* sp. are psychrophiles. This distribution of species strongly suggests that AMD had a more profound affect on biodiversity than the landfill site.

### 4.5. X-ray Fluorescence

The results of the XRF analysis showed large differences in metal concentrations across the site that could potentially be linked to either the acid mine drainage from the coal mine or to the leachate from the landfill site itself. Some distinct trends can be drawn from the XRF data. The silicon and potassium concentrations dropped in Zone E. The iron, calcium, magnesium, sulphur, strontium, barium, cobalt, arsenic and praseodymium concentrations all spiked or increased in Zone E. The phosphorus, manganese, zinc, platinum, nickel and copper concentrations all spiked or increased in Zone F. The aluminium, titanium, chromium, chloride, lead and uranium were approximately the same across all the zones.

#### 4.5.1. Calcium

One of the non-biodegradable wastes that was mentioned specifically [55,56] was gypsum, a mineral composed of calcium sulphate dehydrate (CaSO_4_·2H_2_O) that is soluble in water over time and typically comes from construction materials, such as drywall. When exposed to water, gypsum dissolves, releasing calcium and sulphates into solution. The sulphates may be reduced to produce hydrogen sulphide gas (H_2_S) via sulphate reducing bacteria [63]. Additional calcium has been found to increase the soil pH as gypsum can also be used as a soil treatment agent and fertiliser to increase the pH and calcium levels [64]. This would account for the relative neutrality of the downstream Zone F sites. It may also account for the minor increase in pH in the downstream Zone E group, which did not receive direct landfill influence via surface water but may have been affected by groundwater. As the bypass river was very acidic to start with (pH 3–3.5), the change to a pH of 4–5 could be caused by proximity to the landfill site. Calcium in the downstream Zone E group may also have been more concentrated due to the lack of surface water, whereas the downstream Zone F group had large amounts of water that could have reduced the concentration observed.

#### 4.5.2. Magnesium

In the soils with low magnesium levels, due to the addition of gypsum and excess calcium, the Ca^2+^ can outcompete the other K^+^ and Mg^2+^ cations, resulting in leaching of these cations, and therefore, a reduction in the soil concentration [65]. There was also a high abundance of plant life in the downstream Zone F group.

#### 4.5.3. Potassium

It has been estimated that around 90–98% of soil potassium is unavailable with only 2% ‘free’ potassium [66]. This would account for the very high levels of potassium at all sites. The levels of potassium in the downstream Zone E group were lower, which were likely affected by the very high calcium levels. 

#### 4.5.4. Iron

The iron levels in and around the landfill site were high. There was clear evidence of the influence of iron moving from the mine with a sudden increase in the iron concentrations from sample site U2 (this increased after sample point U6 where the acid mine drainage met the stream). Iron could also be seen on the soil directly as an orange streaks. The pH of the site was also quite acidic in places, which could increase the solubility of iron oxide [67].

**Figure 6:**
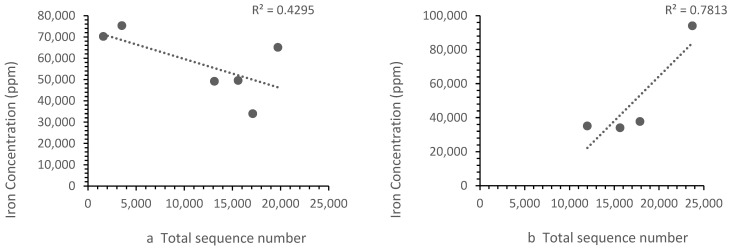
Correlation between (**a**) Zones B, D and E iron concentrations vs. the sequence number, where the sequence number increased as the iron concentration falls and (**b**) the Zone F (i.e., landfill leachate) iron concentration vs. the sequence number, where the sequence number increased with iron concentration. Each point represents a sample site. For iron, the relationship differed in the landfill leachate.

**Figure 7:**
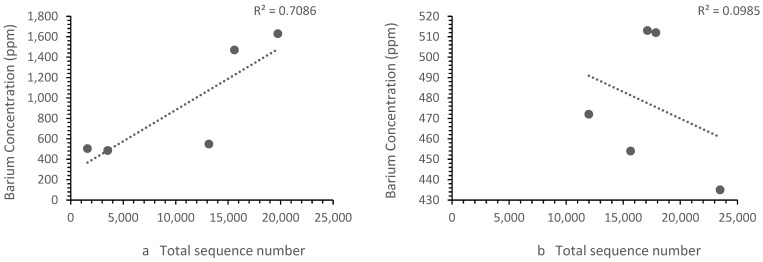
Correlation between (**a**) Zones B, D and E barium concentrations vs. the sequence number, where the sequence number increased as the barium concentration increased and (**b**) the Zone F (i.e., landfill leachate) barium concentration vs. the sequence number, where the sequence number decrease with the barium concentration. Each point represents a sample site. For barium, the relationship differed in the landfill leachate.

The patterns of ion concentration vs. the bacterial diversity differed for different ions. The calcium, zinc and strontium showed the same trend upstream and downstream. However, the iron (Figure 6) and barium (Figure 7) showed a different trend upstream and downstream.

Sample site D11 had higher concentrations of some elements, such as manganese, iron and zinc (Figure 5). This may have been due to the location of D11 near the end of the outflow stream where the flow rate markedly decreased, which could lead to deposition and build-up in the sediment.

### 4.6. Organic Chemistry

The levels of organic carbon in the soils showed wide variation, with the highest (at 12.3%) at site U2 (Zone B) and lowest at sites U6-D4 (Zone D and E). This could either reflect higher bacterial consumption rates or a lower overall biomass. Importantly, it suggests that if significant levels of organic carbon were leached from the landfill site, they were being broken down and not accumulated.

In total, 14 major organic compounds were detected (Table 9). Site U4 contained all the organic compounds detected (suggesting inefficient degradation), whereas only octadecane could be detected at D4 (suggesting relatively efficient degradation). Overall, the downstream site samples had lower levels of organic diversity compared to the upstream samples, although chemical hotspots in terms of the hydrocarbon composition were apparent. Again, the lack of accumulation and lower diversity suggest their active removal.

## 5. Conclusions

The landfill site biology and chemistry differed markedly across the site. This suggested that the landfill site (and the underlying presence of AMD) had a noticeable impact on the environment in Svalbard. The bacterial biodiversity was high both up and downstream, with a lower diversity at the site of the AMD ingress. Along with the fact that similar groups dominated the upstream and downstream communities (away from the direct AMD impact), this suggested that intervention through bioremediation could be effective, since the leachate appeared to allow microbial growth and organic material degradation. Upward and downward spikes in the concentrations of the key groups of inorganic and organic chemicals across the landfill site were observed. The variation in the total organic carbon, observation of biofilms and DAPI counts, taken together, suggested that active growth within the ecosystem was occurring. 

According to these observations, a number of active intervention options would be possible at the site (as elsewhere e.g., [68]). Intervention with bioremediation would likely enhance environmental recovery. A small-scale pilot study could incubate the run-off with isolated bacteria (successfully attempted during this study but not reported here) and develop an optimal intervention strategy to maximise the biodegradation capacity. It remains to be determined which method of bioremediation for this site would be most appropriate (intrinsic, biostimulation or bioaugmentation), although medium-term monitoring could demonstrate lasting recovery over the longer term. A medium-scale pilot study could be used to investigate the effect of chemical alteration (e.g., pH manipulation).

A number of active management plan options are also available: (i) do nothing and allow the environment to recover by itself (risk: damage may continue/escalate), (ii) minor intervention (change aspects of the local environment to favour bioremediation), (iii) medium-scale intervention (an active in-situ bioremediation programme), (iv) full-scale intervention (removal of topsoil to a dedicated site for full-scale industrial bioremediation) and (v) further research to determine the likely timescale and outcomes.

Hence, our overall recommendations would be to (i) develop a strategy for intervention to help the environment recover and (ii) conduct specific experiments to determine the most effective form of bioremediation for this site (intrinsic, biostimulation or bioaugmentation). However, if the site is found to be stable, given the unique combination of chemical and physical gradients in close proximity following significant human intervention in an otherwise pristine and rapidly changing environment, another option might be to protect it as a site of special scientific interest or a microbial biodiversity reserve.

## 6. Summary

Intervention is highly desirable to restore the original environmental conditions due to the extreme nature of the changes in the biodiversity of the microbial community caused by both the leachate from the site and from AMD, as well as the likelihood of significant improvement for low investment and the overall cost-effectiveness of such an intervention. However, the area of impact appears to be relatively small and stable, and the underlying AMD appears to have a more significant impact than the landfill leachate (at least at the superficial level). Changes can be made to enhance the environment and improve restoration by subtly altering the conditions at the site (such as the pH or drainage courses) and by encouraging specific groups of naturally occurring indigenous microorganisms to remove key toxic compounds (particularly the organics). Monitoring of the induced changes is desirable to determine whether the area of influence of the landfill site is stable, receding or increasing over longer time scales and to determine the wider applicability of these observations to acid mine drainage elsewhere in Svalbard.

## Figures and Tables

**Table 1:** The total species number and Shannon diversity index, upstream vs. downstream. Note the lower diversity well upstream of the landfill site and the increased diversity around the AMD influence and downstream. Sites SS5 and SS7 had an order of magnitude lower total species number than the site in general and this reflected the patchy nature of the site. Abbreviations: SS—sample site, U—upstream, D—downstream.

Sample	Species Number	Shannon Diversity Index
SS1 (U 100 m)	1399	1095
SS2 (U 100 m)	1204	989
SS3 (U 100 m)	1328	1011
SS4 (U 200 m)	1621	898
SS5 (U 200 m)	161	866
SS6 (U 200 m)	1297	800
SS7 (D 50 m)	162	1115
SS8 (D 50 m)	1651	1193
SS9 (D 50 m)	1456	1202

**Table 2:** Total relative DAPI counts.

Site	Flow Rate (cm·s^−1^)	Coordinates		DAPI Counts
		**North**	**East**	
D7	50.0	78.17588	015.93550	388
D9	11.9	78.17606	015.93546	320
D11	71.4	78.17616	015.93594	1552
D13	0	78.17675	015.93679	4160
D14	0	78.17662	015.93680	2416
D16	3.0	78.17726	015.94373	1200
D17	0	78.17719	015.94337	2208

**Table 3:** The average pH and temperature at each sample location (acidic sites are denoted in normal text, more neutral are denoted in bold).

Sample	Temperature (°C)		pH	
	Water	Sediment	Water	Sediment
U1 (Zone A)	5.51	5.50	5.81	6.20
U2 (Zone B)	6.35	5.55	5.70	5.70
**U3 (Zone B)**	7.40	9.00	**6.10**	6.40
**U4 (Zone B)**	6.75	6.25	**6.50**	6.50
**U5 (Zone B)**	9.10	7.25	**6.76**	6.80
U6 (Zone D)	11.35	7.95	3.50	3.40
U7 (Zone E)	9.65	8.05	3.00	2.90
D1 (Zone E)	4.05	3.20	5.10	5.70
D2 (Zone E)	10.00	8.40	2.90	3.70
D3 (Zone E)	-	16.25	-	7.60
D4 (Zone E)	8.95	7.50	4.20	4.30
**D5 (Zone F)**	8.75	5.00	**6.40**	6.80
**D6 (Zone F)**	2.00	1.25	**6.70**	6.60
**D7 (Zone F)**	9.55	3.50	**6.60**	6.10
**D8 (Zone F)**	12.70	4.55	**6.10**	5.90
**D9 (Zone F)**	11.65	4.55	**6.80**	6.40
**D10 (Zone F)**	7.55	3.65	**6.70**	6.40
**D11 (Zone F)**	9.45	3.50	**7.10**	6.90

**Table 4:** Sequence number of each genus across each of the sites (only genera including at least one category above >100 sequences at a site were included). The genera in bold were present in the top four most common sequences in at least one of the sites.

Genus	D3	D4	D5	D6	D9	D10	D11	U2	U6	U7
*Acetobacterium*	0	5	142	28	10	1	176	10	0	3
*Acidicapsa*	1	2	4	5	17	1	0	10	250	33
***Acidophilium***	**6**	**6**	**5**	**26**	**102**	**4**	**10**	**15**	**210**	**339**
*Acidocella*	0	17	6	4	514	20	3	5	1	35
*Acidovorax*	149	10	18	100	9	10	97	0	4	0
*Aequorivita*	345	4	37	7	0	1	24	3	1	0
*Afipia*	26	16	54	486	203	6	211	533	2	6
*Algoriphagus*	438	827	393	45	15	1	38	5	2	5
*Alkalibacterium*	38	305	0	0	0	28	5	0	5	0
*Allostreptomyces*	0	0	0	4	0	0	0	1	1	0
*Aminobacter*	26	0	43	23	0	24	116	9	150	0
*Aquaspirillum*	2	3	12	18	126	1	101	14	10	0
*Arenibacter*	137	0	1	0	7	0	5	0	17	4
*Arenibacterium*	9	35	0	0	0	150	3	0	1	0
*Arenimonas*	586	111	59	55	19	2	152	155	2	4
*Aromatoleum*	0	0	17	15	2	174	2	5	1	0
*Arsenicicoccus*	36	5	28	70	5	22	273	35	9	2
*Aureimonas*	16	5	0	4	0	145	5	3	2	0
*Austwickia*	13	3	37	50	8	19	132	26	1	2
*Bdellovibrio*	0	0	1	1	0	154	0	0	2	0
*Beijerinckia*	30	29	18	158	49	21	106	134	1	7
*Bellilinea*	1	0	35	0	2	28	16	10	1	0
*Blastochloris*	1	0	4	134	17	8	39	110	135	3
*Brachybacterium*	1	1	14	7	20	677	30	5	3	0
***Bradyrhizobium***	**88**	**45**	**65**	**849**	**505**	**4**	**448**	**893**	**13**	**14**
***Brevundimonas***	**1561**	**2182**	**306**	**216**	**60**	**5**	**578**	**220**	**1**	**13**
*Caballeronia*	4	2	4	31	41	104	15	36	4	1
*Carnobacterium*	25	122	5	20	2	1	25	0	1	0
*Catenulispora*	4	3	0	108	0	144	2	22	26	0
*Caulobacter*	86	25	114	223	11	1	264	158	3	0
*Cellvibrio*	249	463	3	6	7	4	7	18	1	5
*Citrifermentans*	0	1	82	150	20	72	151	7	1	1
*Clostridium*	138	464	537	114	42	1	428	70	1	15
*Collimonas*	0	0	0	0	1	10	4	0	1	0
*Croceimicrobium*	3	0	0	1	0	232	0	0	135	0
*Cryobacterium*	87	139	74	52	42	1	352	32	1	8
*Cumulibacter*	0	0	0	0	0	0	0	0	1	2
*Cupriavidus*	12	6	29	49	15	7	20	72	127	0
*Curtobacterium*	1	2	0	0	0	237	9	1	215	0
*Curvibacter*	103	38	132	115	30	28	442	89	1	0
*Cypionkella*	122	68	68	43	6	5	183	35	1	4
*Dechloromonas*	2	0	41	97	47	1	621	10	1	0
*Desulfobulbus*	0	0	9	4	0	119	0	0	1	0
*Desulfocastanea*	4	0	141	3	3	1	4	0	0	1
*Desulfopila*	4	0	135	3	4	10	3	0	0	1
*Desulfosediminicola*	5	0	206	5	6	30	6	0	0	1
*Desulfotalea*	5	0	131	6	2	2	8	0	0	2
*Desulfuromonas*	0	0	18	4	0	128	10	3	0	1
*Devosia*	657	428	118	253	27	9	528	100	0	18
*Dokdonella*	97	1	13	17	8	128	43	4	0	0
*Duganella*	30	3	140	133	85	33	319	209	0	3
*Dyella*	19	6	27	1	721	1	16	10	0	1
*Erythrobacter*	106	100	4	20	4	1	33	15	0	0
*Feifantangia*	1	1	0	0	0	27	264	4	0	0
***Flavobacterium***	**2632**	**943**	**2213**	**643**	**131**	**1**	**146**	**1814**	**0**	**0**
*Fuscovulum*	19	20	5	0	2	1	124	2	0	0
*Fusibacter*	2	7	41	210	12	0	4	0	0	0
*Gaetbulibacter*	3	0	0	2	0	245	1	7	0	0
***Gallionella***	**6**	**2**	**52**	**22**	**524**	**8**	**3**	**9**	**0**	**0**
*Gelidibacter*	483	0	6	6	3	0	1	0	0	0
*Gemmobacter*	175	209	36	6	13	0	0	0	0	0
*Geoalkalibacter*	0	0	2	0	0	361	745	48	0	2
*Geobacter*	4	7	327	326	57	0	0	0	0	0
*Glycocaulis*	0	11	0	0	1	0	4	1	0	0
*Halothiobacillus*	3	2	7	4	267	1	0	0	0	0
*Hydrocarboniphaga*	3	0	0	0	0	63	151	26	0	12
***Hydrogenophaga***	**1654**	**1464**	**232**	**480**	**47**	**0**	**0**	**0**	**0**	**0**
*Hyphococcus*	0	0	0	0	0	52	263	161	0	0
*Jannaschia*	26	65	5	0	0	223	427	129	0	9
*Janthinobacterium*	17	10	62	133	41	3	3	24	0	14
*Kineosporia*	0	2	0	165	0	0	0	0	0	0
*Luteibacter*	2	0	0	0	115	1	0	0	0	0
*Lysinimonas*	5	0	0	1	43	230	383	283	0	6
*Lysobacter*	497	85	287	100	40	1	0	0	0	0
*Martelella*	1	1	0	0	0	140	7	161	0	9
*Massilia*	69	10	193	115	180	0	1	0	0	0
*Mesonia*	5	0	0	0	0	59	161	175	0	4
*Mesorhizobium*	465	277	69	188	6	0	0	0	0	0
*Methylocapsa*	3	9	5	104	20	6	4	27	0	0
*Methylococcus*	0	0	7	0	41	189	106	194	0	4
*Methylocystis*	27	93	19	232	51	9	12	18	0	0
*Methylosarcina*	1	1	0	0	0	118	60	127	0	2
*Methylosinus*	17	8	9	143	31	0	0	6	0	0
*Methylotenera*	27	157	15	12	88	15	29	6	0	0
*Micropruina*	0	2	7	0	2	6	110	2	0	0
*Mongoliitalea*	0	0	0	0	0	102	0	96	0	4
*Naasia*	0	2	1	2	0	159	148	57	0	0
*Nitrosococcus*	0	3	0	0	0	6	124	7	0	0
*Nitrosophilus*	0	0	0	0	0	32	110	12	0	2
*Nitrosospira*	0	3	12	4	47	0	50	119	0	0
*Novispirillum*	0	0	5	0	3	122	57	155	0	3
*Ornithinibacter*	0	0	0	0	0	148	154	21	0	0
*Ornithinicoccus*	19	2	30	61	3	207	217	42	0	2
*Oryzicola*	0	2	0	1	1	481	469	63	0	2
*Parabacteroides*	0	0	3	14	5	70	55	128	0	0
*Pedococcus*	2	0	5	4	15	24	190	110	0	0
*Pedomicrobium*	18	7	18	100	12	0	3	4	0	0
*Pelagimonas*	1	0	0	0	0	103	223	13	0	1
*Pelobacter*	2	2	101	99	18	13	21	10	0	0
*Planococcus*	109	253	5	5	1	0	0	0	0	0
*Planomicrobium*	6	102	0	0	7	0	0	1	0	0
***Polaromonas***	**199**	**60**	**305**	**156**	**86**	**620**	**1465**	**77**	**0**	**8**
*Propionivibrio*	1	0	59	49	96	117	82	8	0	1
*Proteiniclasticum*	36	150	76	23	0	0	54	4	0	0
*Pseudomonas*	399	246	49	221	51	117	129	98	0	6
*Pseudorhodoferax*	49	33	72	100	55	125	88	31	0	0
*Pseudoxanthomonas*	123	63	25	3	5	19	27	10	0	4
*Quatrionicoccus*	0	0	5	17	5	15	106	1	0	1
*Ramlibacter*	24	3	34	61	6	748	80	60	0	0
*Rhizobium*	144	80	88	87	3	15	103	28	0	1
***Rhodanobacter***	**22**	**7**	**11**	**0**	**922**	**33**	**5**	**12**	**0**	**0**
***Rhodoferax***	**183**	**278**	**2466**	**1754**	**575**	**2332**	**1454**	**425**	**0**	**20**
*Rhodoplanes*	6	2	5	178	13	28	43	228	0	0
***Sideroxydans***	**4**	**0**	**44**	**20**	**433**	**264**	**42**	**10**	**0**	**4**
*Simplicispira*	22	2	7	44	9	12	215	1	0	0
*Sphingomonas*	118	78	56	105	13	275	237	380	0	2
*Sphingopyxis*	139	11	9	26	0	20	6	8	0	0
*Sphingorhabdus*	161	73	81	11	0	33	184	109	0	5
*Subsaxibacter*	349	1	4	4	2	2	2	5	0	0
*Sulfuricella*	1	1	109	0	131	37	7	0	0	0
*Sulfuricurvum*	0	0	122	1	94	20	2	2	0	5
*Sulfuriferula*	11	23	265	5	515	225	15	5	0	7
*Sulfurimicrobium*	1	1	60	2	182	27	7	2	0	1
*Sulfurimonas*	0	0	232	4	92	21	0	0	0	4
*Sulfurospirillum*	0	0	178	0	18	9	2	2	0	8
*Sulfurovum*	0	0	129	0	0	9	3	0	0	2
*Tabrizicola*	104	248	14	4	4	4	20	11	0	6
*Thermomonas*	435	47	267	177	21	44	311	35	0	5
***Thiobacillus***	**70**	**54**	**1290**	**12**	**191**	**527**	**66**	**7**	**0**	**0**
*Tissierella*	0	0	16	15	5	0	119	0	0	0
*Trichococcus*	30	135	20	5	5	7	25	0	0	0
*Variovorax*	63	29	109	100	41	196	107	64	0	3
*Xanthomonas*	154	13	2	7	5	7	5	2	0	2

**Table 5:** Site specific bacterial distribution in order of the frequency of occurrence (at the species level).

Upstream (Zone B)	Dominant Bacteria
U2	*Flavobacterium xanthum* *Bradyrhizobium canariense* *Flavobacterium sinopsychrotolerans* *Polaromonas soli*
AMD (Zone E)	
U6	*Terracidiphilus gabretensis* *Terriglobus saanensis* *Acidophilum multivorum* *Acidibrevibacterium fodinaquatile*
U7	*Acidophilum multivorum* *Terracidiphilus gabretensis* *Silvibacterium bohemicum* *Occallatibacter savannae*
D3	*Hydrogenophaga taeniospiralis* *Hydrogenophaga palleronii* *Brevundimonas subvibriodes* *Flavobacterium xanthum*
D4	*Brevundimonas subvibrioides* *Brevundimonas denitrificans* *Hydrogenophaga taeniospiralis* *Brevundimonas bullata*
Downstream (Zone F)	
D5	*Rhodoferax ferrireducens* *Thiobacillus thioparus* *Rhodoferax lacus* *Rhodoferax aquaticus*
D6	*Rhodoferax ferrireducens* *Rhodoferax aquaticus* *Rhodoferax lacus* *Bradyrhizobium canariense*
D9	*Gallionella capsiferriformans* *Sideroxydans lithotrophicus* *Rhodanobacter umsongensis* *Rhodanobacter panaciterrae*
D10	*Rhodoferax ferrireducens* *Rhodoferax aquaticus* *Alsobacter soli* *Rhodoferax lacus*
D11	*Polaromonas jejuensis* *Rhodoferax aquaticus* *Rhodoferax sediminis* *Polaromonas glacialis*

**Table 6:** XRF values by element in ppm and location (rounded to the nearest whole number). Values highlighted in bold were the downstream values that appeared to be notably higher than the values from the upstream group. Values highlighted in italics were the downstream values that appeared to be notably lower than the values from the upstream group. Values in parentheses indicate results that did not appear to fit the trend and were treated as spikes.

	U2	U6	U7	D1	D2	D3	D4	D5	D6	D9	D10	D11
Mg	5194	(1870)	5765	**11,660**	(3104)	**15,093**	**9950**	*2078*	*2128*	*1982*	*3566*	(6714)
Al	72,424	73,532	77,879	(68,284)	*37,773*	*38,037*	*23,908*	63,121	51,545	54,772	67,949	63,946
Si	220,630	226,651	230,669	195,476	*117,056*	*103,109*	*57,255*	265,705	189,351	236,391	270,755	195,102
P	476	834	718	*276*	**1463**	*350*	*269*	436	772	822	472	778
S	*4276*	23,218	12,857	10,425	**33,283**	22,593	22,848	10,345	*7657*	11,629	*5620*	*7283*
Cl	326	145	123	142	*0.00*	252	234	325	**596**	**440**	312	154
K	19,051	18,930	19,731	16,645	*9576*	*8728*	*5171*	18,383	15,271	16,011	19,231	16,700
Ca	(6892)	1839	2127	**44,838**	(5335)	**62,487**	**58,620**	**13,192**	**12,805**	**5662**	(3834)	**13,370**
Ti	4310	4383	4484	4875	*3326*	*3622*	*2663*	*3407*	*3268*	*3620*	*3908*	*3642*
Cr	183	244	116	246	*83*	*73*	*46*	**362**	122	137	324	*84*
Mn	418	305	513	467	*192*	*256*	*267*	370	**861**	*203*	*163*	(4270)
Fe	(49,148)	70,235	75,312	**86,363**	112,354	65,099	49,551	*33,967*	*34,039*	*35,109*	*37,774*	(94,022)
Co	87	88	94	139	111	115	90	*49*	*56*	*50*	*46*	98
Zn	109	96	110	128	108	110	113	**161**	**282**	94	**221**	(3189)
As	15	34	33	18	52	31	36	15	14	18	12	33
Sr	195	211	165	**1356**	**319**	**1776**	**1742**	211	245	167	169	**317**
Ba	548	485	504	**1535**	**873**	**1629**	**1470**	513	454	472	512	435
Pr	9	19	11	**31**	21	**44**	**56**	25	26	26	26	*2*
Pt	12	0	5	0	5	0	1	5	3	**22**	**24**	**10**
Pb	22	19	18	17	(4)	27	28	22	27	23	29	20
U	0	0	0	6	(0)	**10**	**8**	0	0	0	1	0
Ni	49	40	39	53	35	35	29	32	45	35	36	**119**
Cu	32	42	36	46	40	48	47	24	33	29	29	**77**

**Table 7:** Total organic carbon concentrations (additional potassium dichromate had to be added to the samples U2, D2, D6, D9 and D11).

Sample	K_2_Cr_2_O_7_ Added (mL)	Ammonium Ferrous Sulphate Used (mL)	% Total Organic Carbon
U2	20.0	24.5	12.3%
U6	10.0	17.2	8.6%
U7	10.0	13.3	6.7%
D1	10.0	16.5	8.3%
D2	20.0	19.5	9.8%
D3	10.0	14.9	7.5%
D4	10.0	12.8	6.4%
D5	10.0	13.8	6.9%
D6	20.0	18.4	9.2%
D9	20.0	18.7	9.4%
D10	10.0	11.1	5.6%
D11	20.0	21.0	10.5%

**Table 8:** Table summarising the compounds, retention time of the compounds and abundance per site. The compounds in italics showed a very restricted distribution.

Retention Time	Compound	D1	D2	D3	D4	D5	U1	U2	U3	U4	U5
21.57	*tetradecane*	0	0	0	0	0	0	0	0	1,089,475	1,089,475
22.38	*pentadecane*	0	0	0	0	0	0	0	19,414	3,807,211	792,581
23.17	hexadecane	0	478,651	0	0	0	108,120	0	821,900	7,010,913	1,683,659
23.92	heptadecane	29,869	87,077	21,570	0	0	154,356	86,294	11,059	8,231,141	1,931,920
24.66	octadecane	3,937,625	851,931	3,900,301	1,652,369	451,665	1,079,014	107,755	1,536,190	14,221,011	2,009,104
25.36	nonadecane	0	0	21,976,999	0	0	0	0	45,733	17,210,268	1,238,436
26.05	2-methylnonadecane	0	0	13,429,150	0	0	249,790	0	880,035	21,281,153	2,478,966
26.71	heneicosane	2,642,633	0	0	0	0	0	0	0	22,515,689	2,072,473
27.37	3-mthylheneicosane	0	0	0	0	0	1,362,985	0	0	23,402,544	2,341,262
28.06	pentacosane	0	0	9,288,967	0	1,156,237	0	0	0	20,210,775	0
28.86	*heptacosane*	0	0	0	0	0	0	0	0	15196749	0
29.68	nonacosane	0	0	16,371,508	0	0	0	0	1,430,870	11,200,263	2,868,845
30.65	hentriacotane	0	0	2,458,057	0	0	0	0	0	7,969,434	0
31.77	*hexatriacotane*	0	0	0	0	0	0	0	0	4,650,755	0

**Table 9:** Greatest bacterial abundance by genus across all the sites.

Genus	U2	U6	U7	D3	D4	D5	D6	D9	D10	D11
*Brevundimonas*	220	1	13	1561	2182	306	216	60	5	578
*Devosia*	100	0	18	657	428	118	253	27	9	528
*Flavobacterium*	1814	0	0	2.632	943	2213	643	131	1	146
*Rhodoferax*	425	0	20	183	278	2466	1754	575	2332	1454

**Table 10:** Greatest bacterial abundance by genus for the Zone B, D and E upstream sites (U2, U6, U7). These sample sites were dominated by acidophilic species (and a low pH).

Genus	U2	U6	U7	D3	D4	D5	D6	D9	D10	D11
*Acidicapsa*	10	250	33	1	2	4	5	17	1	0
*Acidiphilium*	15	210	339	6	6	5	26	102	4	10

**Table 11:** Greatest bacterial abundance by genus for the Zone E downstream sites (D3, D4). These sample sites showed diverse genera, which corresponded to the diverse characteristics of the different sample sites. *Polaromonas* inhibited the environments with extremely low temperatures, whilst the species belonging to genera *Ramlibacter* degraded isoprene and/or were methanotrophic.

Genus	U2	U6	U7	D3	D4	D5	D6	D9	D10	D11
*Polaromonas*	77	0	8	199	60	305	156	86	620	1465
*Ramlibacter*	60	0	0	24	3	34	61	6	748	80
*Thiobacillus*	7	0	0	70	54	1290	12	191	527	66

**Table 12:** Greatest bacterial abundance by genus for the Zone F downstream sites (D5, D6, D9, D10, D11). These sample sites were dominated by mesophilic species. Importantly, they were relatively absent from the acidic sites.

Genus	U2	U6	U7	D3	D4	D5	D6	D9	D10	D11
*Algoriphagus*	5	2	5	438	827	393	45	15	1	38
*Alkalibacterium*	0	5	0	38	305	0	0	0	28	5
*Hydrogenophaga*	0	0	0	1654	1464	232	480	47	0	0

## Data Availability

We are happy to share all data and are in the process of adding to a publically accessible database, in the interim please contact the corresponding author.
